# miR33a/miR33b* and miR122 as Possible Contributors to Hepatic Lipid Metabolism in Obese Women with Nonalcoholic Fatty Liver Disease

**DOI:** 10.3390/ijms17101620

**Published:** 2016-09-24

**Authors:** Teresa Auguet, Gemma Aragonès, Alba Berlanga, Esther Guiu-Jurado, Andreu Martí, Salomé Martínez, Fàtima Sabench, Mercé Hernández, Carmen Aguilar, Joan Josep Sirvent, Daniel del Castillo, Cristóbal Richart

**Affiliations:** 1 Group de Recerca GEMMAIR (AGAUR)-Medicina Aplicada, Institut Investigació Sanitària Pere Virgili (IISPV), Departament de Medicina i Cirurgia, Universitat Rovira i Virgili (URV), Tarragona 43007, Spain; gemma.aragones@iispv.cat (G.A.); alba.berlanga@urv.cat (A.B.); esther.guiu@urv.cat (E.G.-J.); caguilar.hj23.ics@gencat.cat (C.A.); 2 Servei Medicina Interna, Hospital Universitari Joan XXIII Tarragona, Mallafré Guasch, 4, Tarragona 43007, Spain; andreumano@gmail.com; 3 Servei Anatomia Patològica, Hospital Universitari Joan XXIII Tarragona, Mallafré Guasch, 4, Tarragona 43007, Spain; mgonzalez.hj23.ics@gencat.cat (S.M.); jsirvent.hj23.ics@gencat.cat (J.J.S.); 4 Servei de Cirurgia, Hospital Sant Joan de Reus, Departament de Medicina i Cirurgia, Universitat Rovira i Virgili (URV), IISPV, Avinguda Doctor Josep Laporte, 2, 43204 Reus, Tarragona 43204, Spain; fatima.sabench@urv.cat (F.S.); mhernandezg@grupsagessa.com (M.H.); ddelcastillo@grupsagessa.com (D.d.C.)

**Keywords:** miR122, miR33a/b*, nonalcoholic fatty liver disease, obesity

## Abstract

Specific miRNA expression profiles have been shown to be associated with nonalcoholic fatty liver disease (NAFLD). We examined the correlation between the circulating levels and hepatic expression of miR122 and miR33a/b*, the key lipid metabolism-related gene expression and the clinicopathological factors of obese women with NAFLD. We measured *miR122* and miR33a/b* expression in liver samples from 62 morbidly obese (MO), 30 moderately obese (ModO), and eight normal-weight controls. *MiR122* and miR33a/b* expression was analyzed by qRT-PCR. Additionally, miR122 and miR33b* circulating levels were analyzed in 122 women. Hepatic miR33b* expression was increased in MO compared to ModO and controls, whereas *miR122* expression was decreased in the MO group compared to ModO. In obese cohorts, miR33b* expression was increased in nonalcoholic steatohepatitis (NASH). Regarding circulating levels, MO patients with NASH showed higher miR122 levels than MO with simple steatosis (SS). These circulating levels are good predictors of histological features associated with disease severity. MO is associated with altered hepatic miRNA expression. In obese women, higher miR33b* liver expression is associated with NASH. Moreover, multiple correlations between miRNAs and the expression of genes related to lipid metabolism were found, that would suggest a miRNA-host gene circuit. Finally, miR122 circulating levels could be included in a panel of different biomarkers to improve accuracy in the non-invasive diagnosis of NASH.

## 1. Introduction

Nonalcoholic fatty liver disease (NAFLD), the hepatic consequence or precursor of type 2 diabetes and metabolic syndrome, is the most common chronic liver disease in developed countries [1,2,3]. The incidence of NAFLD has increased alarmingly over the last twenty years worldwide. In developed countries, it is between 10% and 30%, and can be as high as between 60% and over 90% among obese subjects [4,5]. It is a multifactorial condition encompassing a wide spectrum of liver damage, ranging from simple steatosis (SS) to nonalcoholic steatohepatitis (NASH). While SS is considered a relatively benign condition with little risk of progression, NASH, may progress to cirrhosis and, in a small percentage of patients, to hepatocellular carcinoma [6]. NASH affects approximately 2%–3% of the general population, increasing to 37% in obese individuals [7,8]. Regarding pathogenesis, adipose tissue and gut, producing and releasing circulating endotoxins, adipokines, and pro-inflammatory cytokines, which contribute to insulin resistance (IR) and free fatty acids accumulation, may induce oxidative stress and hepatocellular damage [9,10,11]. Additionally, among all these “multiple parallel” mechanisms, a deregulation of the lipid metabolism seems to play a crucial role in the pathogenesis of NASH [12].

In recent years, microRNAs (miRNAs) have emerged as important post-transcriptional regulators of lipid metabolism and other pathological conditions [13]. These small endogenous non-coding RNA molecules, embedded in sequences of human genes, seem to act jointly with their host gene products to regulate lipid homeostasis. MiR122, the most abundant miRNA in the liver, seems to be an important factor for the metabolism of glucose and lipids [14]. Additionally, miRNA33a and b* were recently found to be key regulators of such metabolic programs as cholesterol and fatty acid homeostasis [15,16]. Some authors have described altered expression patterns of miRNAs in NAFLD, suggesting that they have a role in the pathogenesis of the disease [17,18,19]. Studies on human NAFLD have identified approximately 90 deregulated miRNAs in NAFLD liver [20,21,22,23,24,25,26,27], and the current view is that they critically contribute to the development and progression of the disease. Furthermore, as nowadays there are no reliable non-invasive biomarkers to distinguish NASH from SS, or fibrosis from NASH, it has been suggested that miRNAs are an ideal class of biomarkers in a variety of pathological conditions, including liver diseases [26], because of their stability in the circulation.

Thus, the aim of the present study was to analyze the liver expression of miR33a/b* and miR122 in women in relation to the degree of obesity and also to the absence/presence of NAFLD. We assessed the relationship between the liver expression of these miRNAs and the expression of the main genes involved in lipid metabolism in these cohorts, and compared the potential of the circulating levels of these miRNAs with the gold-standard method, liver biopsy, as the diagnostic and prognostic biomarkers of differentiation between simple steatosis and steatohepatitis in obese patients.

## 2. Results

Here, we present the main results obtained in response to our four objectives.

### 2.1. Baseline Characteristics of the Subjects

First, we describe the main characteristics of our cohort, including anthropometric, biochemical (glucose and lipid metabolism, liver enzymes) parameters and, finally, parameters of liver histology.

Patients’ baseline characteristics, given in Table 1, show the mean ± SEM of the variables of interest. The cohort was first classified according to the degree of obesity: normal-weight (body mass index (BMI) < 25 kg/m^2^), moderately obese (ModO) (BMI 32–38 kg/m^2^), and morbidly obese (MO) subjects (BMI > 40 kg/m^2^). Biochemical analyses indicate that ModO and MO women had significantly higher levels of homeostatic model assessment 2-insulin resistance (HOMA2-IR), glucose, insulin, HbA1c, and triglycerides than the control group. High-density lipoprotein cholesterol (HDL-C) was significantly lower in the ModO and MO patients than in the control group. However, ModO women had significantly higher levels of fasting glucose than the MO group. Secondly, we classified the ModO and MO cohorts according to the liver pathology into normal liver (NL), simple steatosis (SS) and nonalcoholic steatohepatitis (NASH). Their characteristics are also described in Table 1. Age and anthropometrical measurements were not significantly different between NL, SS and NASH in the obese cohorts. Glucose and HOMA2-IR were significantly higher in both SS and NASH groups than in the NL subjects of the MO group. Our results indicated that aspartate aminotransferase (AST) and alanine transaminase (ALT) activity was higher in both SS and NASH subjects in the MO than in MO patients with NL histology. It is important to note that our results didn’t show statistically differences regarding the presence of NASH according to obesity degree. In fact, NASH affects 40% of ModO patients and 35% in the MO group in our study. However, as expected and it is shown in Table 1, the lobular inflammatory grade was significantly increased in NASH patients of the MO group in comparison to the ModO NASH group.

### 2.2. Evaluation of the Liver Expression of miR33a/b* and miR122 According to Body Mass Index (BMI)

The first objective of the present study was to analyze the liver expression of miR33a/b* and miR122 in women in relation to the degree of obesity. First of all, we want to clarify that, in the present work, we included the study of miR33b* liver expression instead of miR33b because the first accumulates to higher steady-state levels in the liver than miR33b and, moreover, it has been little studied.

**Figure 1:**
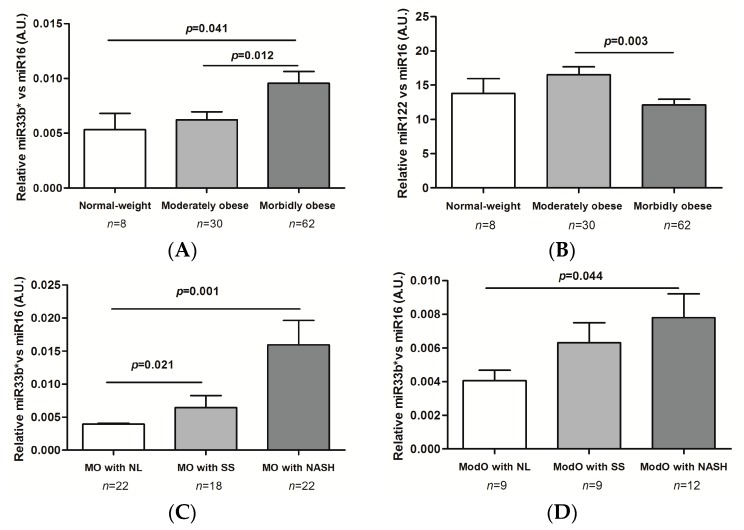
miRNA liver expression according to obesity degree and liver histology. (**A**) Differential expression of miR33b*; and (**B**) *miR122* depending on the degree of obesity; (**C**) miR33b* liver expression in morbidly; and (**D**) moderately obese patients according to liver histology. Morbidly obese (MO) patients with normal liver (NL); MO patients with simple steatosis (SS); MO patients with nonalcoholic steatohepatitis (NASH); Moderately obese (ModO) patients with NL; ModO patients with SS; ModO patients with NASH; AU, arbitrary units. One-way ANOVA was used to compare the gene expression between groups. Results are shown as the mean ± SEM. *p* < 0.05 is considered statistically significant.

Our results indicate that the gene expression of miR33b* was significantly greater in MO women than in ModO and control women (*p* = 0.012 and *p* = 0.041; Figure 1A). However, the gene expression of miR122 decreased significantly in the MO group compared to ModO (*p* = 0.003; Figure 1B). With regard to miR33a, we found no significant differences in gene expression between normal-weight subjects, and the ModO or MO group (*p* = 0.425 and *p* = 0.674).

### 2.3. Evaluation of the Liver Expression of miR33a/b* and miR122 According to Liver Histology

Our second objective was to analyze the liver expression of miR33a/b* and miR122 in women in relation to the absence/presence of NAFLD. Then we present the results divided into obesity groups (first MO and second ModO patients), in order to facilitate the understanding.

#### 2.3.1. Morbidly Obese MO Patients

The gene expression of miR33b* was significantly upregulated in NAFLD compared to NL (*p* < 0.001). Later, we classified the NAFLD cohort into SS and NASH. miR33b* expression was significantly higher in NASH and SS than in NL (*p* = 0.001 and *p* = 0.021; Figure 1C). We found no significant differences in miR33a and miR122 expression.

#### 2.3.2. Moderately Obese ModO Patients

The gene expression of miR33b* was significantly up-regulated in NAFLD compared to NL (*p* = 0.010). Furthermore, the gene expression of miR33b* was significantly higher in NASH than in NL (*p* = 0.044; Figure 1D). There were no significant differences in the expression of miR33a and miR122.

In addition, we explored the association between miRNAs expression in liver and histopathological features. We found that miR33b* correlated positively with hepatocyte ballooning and lobular inflammation (*r* = 0.343, *p* = 0.001; *r* = 0.358, *p* < 0.001).

### 2.4. Evaluation of the Liver Expression of Genes Involved in Lipogenesis, Lipid Oxidation, and Secretion of Lipoproteins According to BMI

In order to answer to our third objective, that is to say to assess the relationship between the liver expression of these miRNAs and the expression of the main genes involved in lipid metabolism in these cohorts, we evaluated the liver expression of genes involved in lipogenesis, lipid oxidation, and secretion of lipoproteins according to BMI and liver histology. We present these results following the same pattern as miRNA expression study.

**Figure 2:**
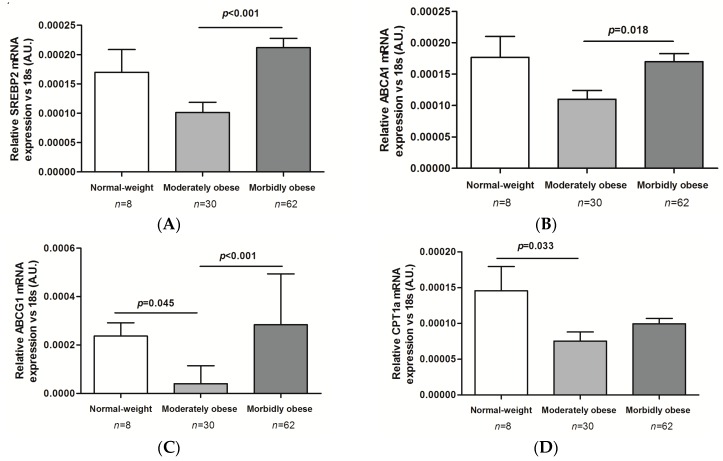
Liver expression of genes involved in lipoprotein secretion (**A**–**C**) and lipid oxidation (**D**) depending on the degree of obesity. *ABCA1*, ATP binding cassette transporters A1; *ABCG1*, ATP binding cassette transporters G1; *CPT1α*, carnitine palmitoyltransferase 1 alpha; *SREBP2*, sterol-regulatory-element-binding protein. Differences between groups were calculated using one-way ANOVA. Results are shown as the mean ± SEM. *p* < 0.05 is considered statistically significant.

The results of the liver expression of genes related to lipid metabolism depending on the degree of obesity are shown in Figure 2A–C. It is noteworthy that the expression of the genes involved in the secretion of lipoproteins was upregulated in MO patients compared to ModO patients sterol-regulatory-element-binding protein 2 (*SREBP2*): *p* < 0.001; ATP binding cassette transporters A1 (*ABCA1*): *p* = 0.018; ATP binding cassette transporters G1 (*ABCG1*): *p* < 0.001, while the expression of the genes involved in lipid oxidation was diminished in both ModO and MO women compared to the normal-weight controls (carnitine palmitoyltransferase 1 alpha (*CPT1α*): *p* = 0.033, carnitine *O*-octanoyltransferase (*CROT*): *p* = 0.003; Figure 2D,E). The results of the normal-weight cohort should be interpreted with caution because of the lower number of liver samples available in this cohort. We found no significant differences in the expression of lipogenic genes according to BMI.

### 2.5. Evaluation of the Liver Expression of Genes Involved in Lipogenesis, Lipid Oxidation, and Secretion of Lipoproteins Based on Liver Histology

#### 2.5.1. MO Patients

*SREBP2* and *ABCG1* gene expression was significantly higher in NAFLD than in NL (*p* = 0.034 and *p* = 0.021, respectively). Furthermore, gene expression of *SREBP2* was significantly higher in NASH vs. NL (*p* = 0.028; Figure 2F) and *ABCG1* was significantly up-regulated in NASH vs. SS and vs. NL (*p* = 0.019 and *p* = 0.006; Figure 2G). The expression of the other genes studied showed no significant differences between groups.

#### 2.5.2. ModO Patients

**Figure 3:**
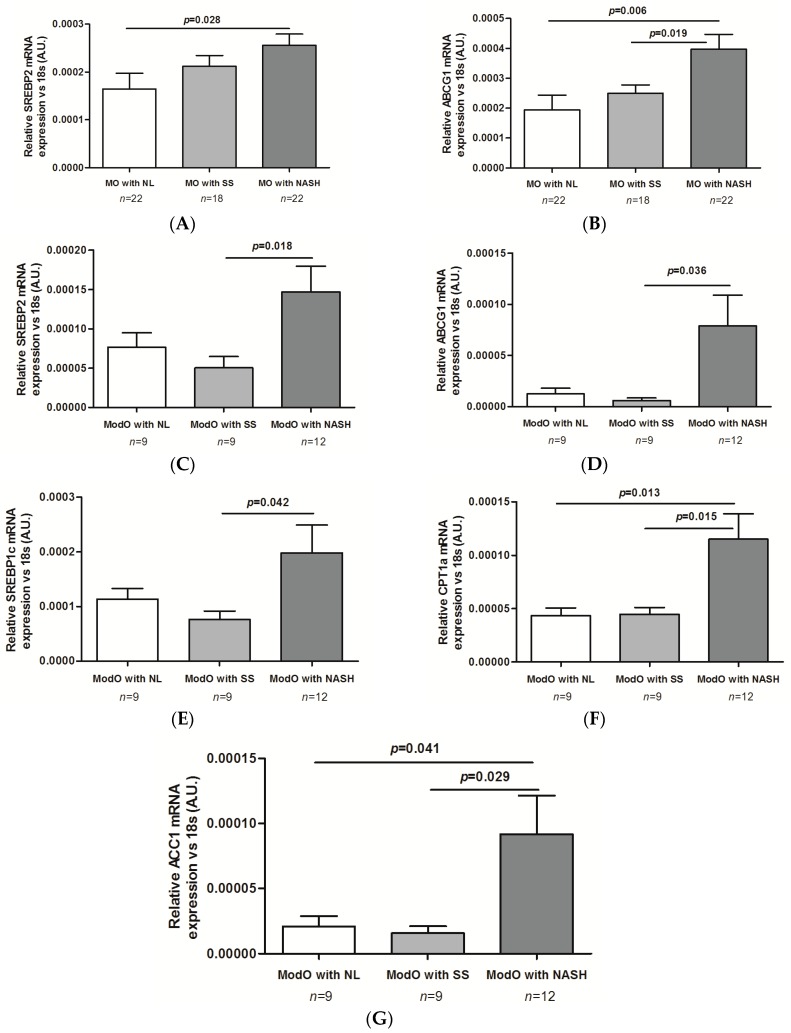
Liver expression of *SREBP2* (**A**) and *ABCG1* (**B**) in morbidly obese patients according to liver histology and liver expression of genes involved in lipoprotein secretion (**C**–**E**); lipogenesis (**F**) and lipid oxidation (**G**) of moderately obese patients according to liver histology. Morbidly obese (MO) patients with normal liver (NL); MO patients with simple steatosis (SS); MO patients with nonalcoholic steatohepatitis (NASH); Moderately obese (ModO) patients with NL; ModO patients with SS; ModO patients with NASH; AU, arbitrary units. Differences between groups were calculated using one-way ANOVA. Results are shown as the mean ± SEM. *p* < 0.05 is considered statistically significant.

*SREBP2* and *ABCG1* gene expression (genes related to lipoprotein secretion) were significantly up-regulated in NASH vs. SS (*p* = 0.018 and *p* = 0.036; Figure 3A,B). Furthermore, *SREBP1c* and *ACC1* gene expression (genes related to liver lipogenesis) was higher in NASH vs. SS (*p* = 0.042 and *p* = 0.029). *ACC1* gene expression was also increased in NASH vs. NL (*p* = 0.041; Figure 3C,D). Lastly, as far as fatty acid oxidation is concerned, we only found increased *CPT1α* gene expression in NASH vs. NL and vs. SS (*p* = 0.013 and *p* = 0.015; Figure 3E).

### 2.6. Significant Correlations of the miR33 a/b* and miR122 Expression with the Expression of Genes Involved in Lipid Metabolism

**Figure 4:**
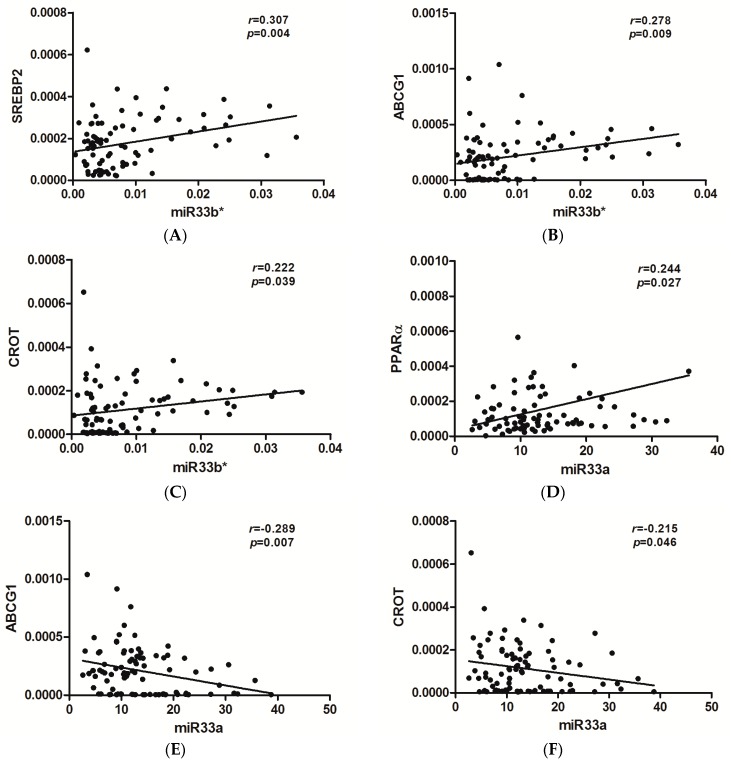
Correlation of the liver expression of miR33b* (**A**–**C**) and *miR33a* (**D**–**F**) with the liver expression of genes involved in lipid metabolism: lipogenesis, fatty acid oxidation and hepatic secretion of lipoproteins. *SREBP2*, sterol-regulatory-element-binding protein; *ABCG1*, ATP binding cassette transporters G1; *CROT*, carnitine *O*-octanoyltransferase; *PPARα*, peroxisome-proliferator-activated receptor α. The strength of association between variables was calculated using Spearman’s r correlation test. *p* < 0.05 is considered statistically significant.

In Figure 4 we showed significant correlations between miRNAs and the expression of genes related to lipid metabolism. We obtained positive correlations of miR33b* gene expression with the mRNA expression of genes related to lipoprotein secretion (*SREBP2* and *ABCG1*) and a gene related to lipid oxidation (*CROT*). We also found a positive correlation between the liver expression of *miR33a* and *PPARα*. However, we found negative correlations between the expression of this miRNA, and *ABCG1* and *CROT*. Finally, we found no correlation between *miR122* expression and the expression of genes involved in lipid metabolism.

### 2.7. Evaluation of miRNA Circulating Levels of miR33b* and miR122 According to BMI

The last objective of our work was to study the potential of the circulating levels of these miRNAs as diagnostic and prognostic biomarkers of differentiation between simple steatosis and nonalcoholic steatohepatitis. We also evaluated them according to BMI and to liver histology.

No significant differences were found in the hepatic expression of *miR33a* according to degrees of obesity and liver histology so we no longer determine their circulating levels.

**Figure 5:**
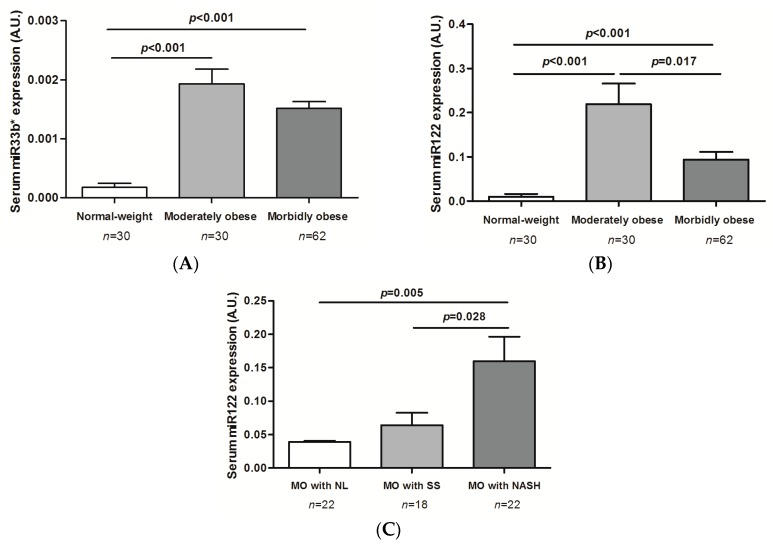
Circulating miR33b* (**A**) and miR122 (**B**) expression depending on the degree of obesity. (**C**) Differential circulating miR122 expression in morbidly obese patients according to liver pathology. MO with NL, morbidly obese patients with normal liver; MO with SS, morbidly obese patients with simple steatosis; MO with NASH, morbidly obese patients with steatohepatitis; AU, arbitrary units. Differences between groups were calculated using one-way ANOVA. Results are shown as the mean ± SEM. *p* < 0.05 is considered statistically significant.

Circulating miR33b* levels were higher in ModO and MO women than in normal-weight subjects (*p* < 0.001; Figure 5A). We found significant differences in the circulating levels of miR122 between normal-weight subjects with ModO and the MO group (*p* < 0.001). Moreover, circulating levels of miR122 were significantly higher in patients with ModO than in those with MO (*p* = 0.017; Figure 5B).

### 2.8. Evaluation of miRNA Circulating Levels of miR33b* and miR122 According to Liver Histology

#### 2.8.1. MO Patients

MO patients with NAFLD had significantly higher miR122 circulating levels than MO with NL (*p* = 0.006). Moreover, the most important result was that MO patients with NASH showed significantly higher circulating levels of miR122 than MO with SS (*p* = 0.028; Figure 5C). Additionally, circulating levels of miR122 were significantly higher in MO NASH than in MO NL women (*p* = 0.005; Figure 5C). However, we found no significant differences in miR33b* circulating levels between the groups classified by liver histology.

#### 2.8.2. ModO Patients

There were no significant differences in circulating levels of miR33b* and miR122 between the groups classified by liver histology in Mod O women.

### 2.9. Correlations between Circulating Levels of miRNA and Its Expression in Liver

When we studied the correlation between miRNAs and their expression in liver, a significant correlation was detected between serum and hepatic *miR122* expression (*r* = 0.253; *p* = 0.019). We found no correlation between serum levels and hepatic expression of the other miRNAs studied.

### 2.10. Correlations of miRNA Circulating Levels with Biochemical Parameters and the Stratification of the Severity of Histological Disease

miRNA 122 circulating levels revealed a strong positive correlation with glucose (*r* = 0.430, *p* < 0.001) and negative correlation with HDL-C (*r* = −0.305, *p* = 0.001). A significant correlation was also observed between miR122 and liver enzymes (AST: *r* = 0.367, *p* < 0.001; ALT: *r* = 0.351, *p* < 0.001). The correlation of miR33b* circulating levels with glucose, triglycerides and AST was also positive (glucose: *r* = 0.214, *p* = 0.025; *p* = 0.004; TG: *r* = 0.279; *p* = 0.004; AST: *r* = 0.203, *p* = 0.046; Table S1). However, there is a negative correlation between miR33b* circulating levels and HDL-C (*r* = −0.276; *p* = 0.004).

We also explored the association between the miRNA circulating levels and the histological severity of NAFLD. We found a positive correlation between miR122 and lobular inflammation and hepatocellular ballooning (*r* = 0.225, *p* = 0.017; *r* = 0.200, *p* = 0.035; Table S1).

### 2.11. Evaluation of miR122 Circulating Levels as a Biomarker of Nonalcoholic Fatty Liver Disease (NAFLD)

This study evaluated the diagnostic efficacy of miR122 circulating levels as a biomarker of NAFLD in a group of patients with liver histology indicative of NAFLD. A cutoff point and the area under the curve were determined so that NAFLD could be diagnosed. To evaluate the extent to which these miRNAs can predict histological features associated with disease severity (hepatocellular ballooning, lobular inflammation), receiver operating characteristic (ROC) curves were obtained. The accuracy with which miR122 discriminates NAFLD from non-NAFLD subjects and an advanced disease from a mild clinical form showed an average area under the curve of receiver operating characteristics (AUROC) of about 0.82 and 0.76, respectively (Table 2).

The binary logistic regression model that contained miR122 serum levels adjusted for age, BMI, HDL-C, triglycerides, AST, and ALT as covariates showed that miR122, along with ALT, made the highest contribution to the detection of the hepatocellular ballooning presence in population studied and was expressed by the exponent values of the beta coefficients (exp (B) or odd ratio: 2.19 for miR122; 1.03 for ALT; Table S2). In fact, the overall model explained 38% of the variability in hepatocellular ballooning in liver.

## 3. Discussion

In a previous study, we demonstrated a clear relationship between NAFLD and genes related to the de novo synthesis of fatty acids in a cohort of women with MO [12]. Considering that miRNA liver expression is different in patients with NAFLD, and that this may be related to the gene expression of hepatic lipid metabolism, the current case-control study aimed to go a step further by exploring the role of miRNAs in obese patients with NAFLD at different severities of histological disease. We examined the correlation between the hepatic expression of *miR122* and miR33a/b*, the key lipid metabolism-related gene expression, and the clinicopathological factors of ModO and MO patients with NAFLD. We also investigated whether circulating miRNA levels are good predictors of NAFLD severity.

Nowadays, the relationship between human liver miRNA expression and degrees of obesity has not been studied yet. The present work provides evidence that morbid obesity is associated with altered hepatic miRNA expression in humans, specifically the up-regulation of miR33b* and the down-regulation of *miR122*. In this sense, recent studies report the functional involvement of miRNAs in metabolic and endocrine pathways and adipogenesis [28,29]. The differential expression of miRNAs has been reported in the adipose tissues of obese vs. non obese subjects [30,31,32,33,34,35,36]. Additionally, circulating levels of miRNAs are dysregulated in men with MO and this expression signature can change with extensive weight loss [37].

Although there are numerous studies on the role of miRNAs in viral hepatitis and hepatocellular carcinoma in humans [13], relatively few of them focus on modulating the pathogenesis of NAFLD. In this respect, although miR33b* expression has been found in several human tissues including the liver [16], its relationship with the presence of NAFLD has not been previously described in a human cohort. Therefore, our study establishes for the first time a clear relationship between NALFD/NASH and higher miR33b* liver gene expression in both ModO and MO women.

Another finding is that there were no significant differences in *miR33a* and *miR122* expression between groups classified by liver histology. However, some authors have reported that liver *miR122* expression is decreased in NASH [20,26] and miR33a was increased in NASH individuals [38]. These discrepancies might be partially explained by differences in the cohort of patients studied. The subjects included in the first and second cohort were, respectively, moderately and mild obese men and women. The third cohort was Mexican mestizo subjects with morbid obesity and a large range aged.

Some authors have previously established that *miR33a* and *miR33b* work in concert with their host genes, *SREBP2* and *SREBP1*, to ensure that a cell’s metabolic state is balanced in different in vitro studies [39,40]. In our study, to gain further insight into the relationship between miRNAs and hepatic lipid metabolism alterations, we first evaluated the liver expression of genes related to lipid metabolism in an obese cohort. In MO women, only *ABCG1* expression was increased in NASH vs. SS. In ModO patients, *SREBP1c* and *ACC1* expression, genes related to liver lipogenesis, *SREBP2* and *ABCG1*, genes related to lipoprotein secretion, and *CPT1α*, related to fatty acid oxidation, were increased in NASH vs. SS. In addition, we found a positive correlation between *miR33a* and *PPRAα* expression and a negative correlation with *ABCG1* and *CROT*. In this respect, *miR33a* and *miR33b*, intronic miRNAs located in the *SREBP2* and *1* genes, respectively, have recently been shown to regulate lipid homeostasis in concert with their host genes [39,40,41,42]. Moreover, Goedeke et al., investigated the functional role of *miR33a* and *miR33b* and their passenger strands, miR33a*/b*, and demonstrated that they share a similar lipid metabolism target gene network. They also showed that *miR33* and miR33* represses key enzymes involved in cholesterol efflux (ABCA1 and NPC1) and fatty acid metabolism (CROT and CPT1α) in human hepatocyte cell lines [16], in agreement with our findings. Accordingly, Vega-Vadillo et al. [38], in a MO cohort, have described that the relative expression of *miR33a* correlated inversely with ABCA1 protein levels. Moreover, *miR33* target gene, *CROT*, correlated inversely with *miR33a*. However, regarding miR33b*, in our study, we found positive correlations between this miRNA and genes related to lipoprotein secretion and to lipid oxidation. These discrepancies could be explained because our study is conducted in a cohort of obese women, whereas Goedeke et al. studied this relationship in human hepatic cell line [16]. It is known that a single miRNA can have multiple targets, potentially providing simultaneous regulation of the genes involved in a physiological pathway. Moreover, several different miRNAs can act synergistically and additively at multiple target sites of single miRNA [43]. Therefore, as it seems to exist important interaction networks between miRNAs and mRNA that can be further expanded by feedback or feed-forward loops, the effect of a single miRNA may be difficult to detect or interpret [44]. Altogether, these data reveal an integrated miRNA-host gene circuit governing lipid metabolism with possibly important therapeutic implications.

miRNAs can also circulate freely in blood, so we evaluated the microRNA circulating levels of miR33b* and miR122 in our cohort. We detected a correlation between serum and hepatic miR122 expression, in agreement with other authors [45], which suggests that miR122 released from hepatic cells enters the bloodstream. However, Pirola et al. described an inverse relationship between the circulating and tissue expression of *miR122*, and suggested that the lower expression of *miR122* in liver is a consequence of a high rate of release into the circulation [26].

Of particular interest among our findings is that circulating miR122 and miR33b* levels were higher in ModO and MO women than in normal-weight subjects. Moreover, circulating levels of miR122 were higher in patients with ModO than in those with MO. In this respect, previous studies have shown that several miRNAs seem to be involved in the regulation of adiposity and insulin sensitivity. Wang et al. showed that elevated circulating miR122 was positively associated with obesity and insulin resistance in young adults [46]. Other authors have reported that circulating miRNA are deregulated in severe obesity and that bariatric surgery-induced weight loss leads to marked changes in this profile [37].

Although liver biopsy is still the *gold standard* for the histopathological diagnosis of NAFLD, its invasive nature means that it is difficult to use and unsatisfactory. Therefore, it is essential to search for NASH biomarkers. To do so, we examined circulating miRNAs and their association with the stratification of histological disease severity, and we found that miR122 levels were increased in MO patients with NAFLD compared with MO with NL. It should also be noted that MO women with NASH showed significantly higher circulating levels of miR122 than MO with SS. Similarly, Pirola et al. [26] analyzed 84 circulating miRNAs and observed the most dramatic and significant changes in the miR122 serum levels. Other authors described higher serum levels of miR122, among others, in NAFLD patients and also that miR122 serum levels were correlated with the severity of liver steatosis [47,48]. Moreover, in a study of patients with chronic hepatitis C and NAFLD, Cermelli et al. found that plasma levels of miR122 were higher than in healthy controls. Interestingly, levels of miR122 were positively correlated with disease severity from SS to steatohepatitis [49]. As the role of circulating miR122 in predicting liver damage has been replicated in liver diseases of different etiologies, circulating miR122 has also been proposed as an early disease severity-dependent biomarker of liver injury because its levels increase before those of serum ALT [50,51].

We also explored the association between miRNA circulating levels and the severity of NAFLD histology and found a positive correlation between miR122 and lobular inflammation and hepatocellular ballooning. ROC analysis revealed that miR122 has the potential to distinguish NASH from SS, because it predicts hepatocellular ballooning or lobular inflammation with an AUROC of 0.76. Although this predictive value is not good enough for an ideal biomarker, the ability of miR122 to predict NASH is quite similar to that of other studies [52,53,54]. However, as these authors suggested, compared to single biomarkers, the stepwise combination of different biomarkers can further improve the accuracy of diagnosing NASH.

Our cohort has made it possible to establish clear relationships between obese women with NAFLD and altered hepatic miRNA expression, without the interference of such confounding factors as gender or age. However, these results cannot be extrapolated to men or overweight subjects.

## 4. Materials and Methods

### 4.1. Subjects

The study was approved by the institutional review board (Comitè Ètic d’Investigació Clínica, Hospital Universitari Joan XXIII de Tarragona, 04p/2014, 21 February 2014). All participants gave written informed consent for participation in medical research. In this study we included 62 morbidly obese women (BMI > 40 kg/m^2^) (MO), 30 moderately obese women (BMI 32–38 kg/m^2^) (ModO), and 30 normal-weight control women (BMI < 25 kg/m^2^). Liver biopsies were obtained from the morbidly obese women during planned bariatric surgery, from the moderately obese women during laparoscopic cholecystectomy for benign gall bladder disease or laparoscopic hiatus hernia repair and from the normal-weight women by means of percutaneous biopsy. Of 30 normal-weight control women, we only obtained liver biopsies from 8 women. All biopsies were performed for diagnostic indications. The pathology service of the hospital provided the control liver samples, taken with informed consent, and once they had been shown to be normal, the minimal excess sample was used to perform gene expression analysis.

The weight of all subjects was stable for at least three months prior to surgery. The exclusion criteria, biochemical analysis, and NAFLD diagnosed were described previously [12]. Liver samples were scored by experienced hepatopathologists using the methods described elsewhere [55,56].

According to their liver pathology and BMI, patients were sub-classified into the following groups: (1) Liver controls: normal-weight subjects with normal liver (NL) histology (*n* = 8); (2) MO with NL histology (*n* = 22); (3) MO with simple steatosis (SS) (micro/macrovesicular steatosis without inflammation or fibrosis, *n* = 18); (4) MO with nonalcoholic steatohepatitis (NASH) (Brunt grade 1–3, *n* = 22); (5) ModO with NL histology (*n* = 9); (6) ModO with SS (micro/macrovesicular steatosis without inflammation or fibrosis, *n* = 9); and (7) ModO with NASH (Brunt grade 1–3, *n* = 12).

### 4.2. RNA Isolation and Real-Time PCR

The liver samples obtained were conserved in RNAlater (Sigma, San Louis, MO, USA) for 24 h at 4 °C and then stored at −80 °C. TaqMan Assays predesigned by Applied Biosystems (Foster City, CA, USA) were used for the detection of *LXRα*, *SREBP1c*, *ACC1*, *FAS*, *PPARα*, *CPT1α*, *CROT*, *SREBP2*, *ABCA1*, *ABCG1*, and *18S* ribosomal RNA that was used as a housekeeping gene. The total RNA was isolated in accordance with the manufacturers’ protocols RNeasy Mini kit (Qiagen, Barcelona, Spain). cDNA was synthesized using a High Capacity RNA-to-cDNA Kit (Applied Biosystems).

To quantify miRNA from liver samples, we used the miRNeasy Mini Kit (Qiagen, Hilden, Alemania) for the isolation of total RNA. The specific primers used—hsa-miR33a, hsa-miR33b*, hsa-miR122—were all from Applied Biosystems, and values were normalized with the housekeeping gene hsa-miR-16.

To quantify miRNA from serum samples, we used the miRNeasy Serum/Plasma Kit (Qiagen). The housekeeping gene C. *elegans* miR39 miRNA mimic was added to the serum samples before RNA was extracted (miRNeasy Serum/Plasma Spike-In Control).

All reactions were carried out in duplicate in 96-well plates using the 7900HT Fast Real-Time PCR systems (Applied Biosystems).

### 4.3. Statistical Analysis

All the values reported are expressed as mean ± SEM and were analyzed using SPSS version 23.0 (IBM Corporation, New York, NY, USA). Differences between groups were calculated using Student’s *t*-test or one-way ANOVA. The strength of association between variables was calculated using Pearson’s method or Spearman’s ρ-correlation test. Area under the receiver operating characteristic curve (AUROC) was used as an accuracy index for evaluating the diagnostic performance of the selected miRNA. To analyze the potential of circulating miRNAs to designate hepatocellular ballooning in liver, we performed a binary logistic regression model. miR122, with a non-normal distribution, were log transformed for this analysis. *p* values <0.05 were considered to be statistically significant.

## 5. Conclusions

In conclusion, there is also a clear relationship between the presence of NASH and higher miR33b* liver expression in both ModO and MO women. Moreover, miR33a/b* seem to act as “micro-controllers” of hepatic lipid metabolism in NAFLD. Finally, the circulating levels of miR122 could be included in a panel of different biomarkers to improve the accuracy of the non-invasive diagnosis of NASH.

## Figures and Tables

**Table 1:** Clinical characteristics of the study cohort classified according to the BMI and histopathological characteristics.

Variables	Normal-Weight (*n* = 30) Mean ± SEM	Moderate Obesity (*n* = 30) Mean ± SEM			Morbid Obesity (*n* = 62) Mean ± SEM		
	–	NL (*n* = 9)	SS (*n* = 9)	NASH (*n* = 12)	NL (*n* = 22)	SS (*n* = 18)	NASH (*n* = 22)
Age (years)	41.0 ± 2.2 *^,£^	49.8 ± 3.7	49.06 ± 5.2	52.23 ± 10.5	46.3 ± 2.6	47.2 ± 2.4	48.8 ± 2.5
Weight (kg) ^§^	58.2 ± 1.5 *^,£^	90.4 ± 2.5	96.5 ± 4.0	89.1 ± 2.6	120.3 ± 3.8	121.1 ± 4.5	119.8 ± 3.1
WC (cm) ^§^	75.2 ± 2.1 *^,£^	107.8 ± 1.8	111.0 ± 3.8	107.2 ± 3.8	132.4 ± 5.6	132.3 ± 4.3	132.6 ± 3.0
BMI (kg/m^2^) ^§^	22.1 ± 0.4 *^,£^	35.4 ± 0.7	36.2 ± 0.8	35.1 ± 0.9	48.5 ± 1.6	48.9 ± 2.1	47.2 ± 1.0
Glucose (mg/dL) ^§^	83.3 ± 2.0 *^,£^	140.9 ± 22.8	131.8 ± 25.5	161.8 ± 25.5	88.5 ± 3.0	126.4 ± 5.9 ^#^	128.8 ± 7.2 ^#^
Insulin (mUI/L)	6.1 ± 0.6 *^,£^	10.7 ± 2.8	15.2 ± 3.9	26.7 ± 9.4	12.2 ± 1.6	19.1 ± 2.9	27.6 ± 6.9
HbA1c (%)	4.7 ± 0.1 *^,£^	6.7 ± 0.8	5.3 ± 0.2	7.5 ± 1.1	5.1 ± 0.1	6.1 ± 0.3	5.9 ± 0.4
HOMA2-IR	0.8 ± 0.1 *^,£^	1.39 ± 0.4	2.5 ± 0.8	3.6 ± 1.6	1.5 ± 0.2	2.6 ± 0.37 ^#^	3.6 ± 0.8 ^#^
HDL-C (mg/dL)	55.5 ± 2.0 *^,£^	39.7 ± 4.5	44.6 ± 2.4	38.2 ± 2.9	44.6 ± 2.4	37.4 ± 2.4	39.1 ± 1.6
LDL-C (mg/dL)	103.7 ± 3.5	103.3 ± 14.1	101.8 ± 8.2	105.5 ± 20.8	98.1 ± 7.2	106.2 ± 6.9	105.5 ± 6.5
Triglycerides (mg/dL)	87.2 ± 9.9 *^,£^	109.6 ± 17.9	134.9 ± 19.9	197.3 ± 37.6	133.8 ± 11.2	177.1 ± 16.5	185.0 ± 19.4
AST (U/L)	22.3 ± 1.5 *^,£^	27.7 ± 5.4	47.7 ± 9.6	52.0 ± 12.8	19.7 ± 1.2	43.0 ± 8.4 ^#^	52.3 ± 7.6 ^#^
ALT (U/L)	21.5 ± 2.9 *^,^^£^	28.1 ± 7.7	36.7 ± 7.6	48.1 ± 10.2	19.2 ± 1.2	43.6 ± 6.1 ^#^	54.2 ± 6.9 ^#^
GGT (U/L)	22.2 ± 6.3 ^£^	30.4 ± 15.5	20.6 ± 5.5	25.4 ± 5.2	21.4 ± 6.5	34.6 ± 3.9	41.2 ± 7.0
ALP (U/L)	61.2 ± 4.5	72.2 ± 13.3	63.2 ± 5.1	67.2 ± 6.2	64.4 ± 3.4	67.3 ± 4.2	74.5 ± 2.9
**Steatosis grade**			1.33 ± 0.2	1.42 ± 0.1		1.94 ± 0.2 ^‡^	1.72 ± 0.1
Mild (1)	–	–	6 (67%)	7 (58%)	–	6 (33%)	9 (41%)
Moderate (2)	–	–	3 (33%)	5 (42%)	–	7 (39%)	10 (45%)
Severe (3)	–	–	–	–	–	5 (28%)	3 (14%)
**Lobular inflammatory grade**			0.11 ± 0.1	2.10 ± 0.1		0.17 ± 0.1	1.36 ± 0.1 ^†^
Absence (0)	–	–	8 (89%)	–	–	15 (83%)	–
Mild (1)	–	–	1 (11%)	1 (8%)	–	3 (17%)	16 (73%)
Moderate (2)	–	–	–	9 (75%)	–	–	4 (18%)
Severe (3)	–	–	–	2 (17%)	–	–	2 (9%)
**Hepatocellular ballooning**			0.11 ± 0.1	1.00 ± 0.1		0.17 ± 0.1	1.09 ± 0.1
Absence (0)	–	–	8 (89%)	1 (8%)	–	15 (83%)	–
Mild (1)	–	–	1 (11%)	10 (84%)	–	3 (17%)	20 (91%)
Moderate (2)	–	–	–	1 (8%)	–	–	2 (9%)
Severe (3)	–	–	–	–	–	–	–

ALT, alanine aminotransferase; ALP, alkaline phosphatase; AST, aspartate aminotransferase; BMI, body mass index; GGT, gamma-glutamyltransferase; HbA1c, glycosylated haemoglobin; HDL-C, high-density lipoprotein cholesterol; HOMA2-IR, homeostatic model assessment 2-insulin resistance; LDL-C, low-density lipoprotein cholesterol; SS, simple steatosis; NASH, steatohepatitis; NL, normal liver; WC, waist circumference. Data are expressed as mean ± SEM or number of cases (%). Differences between groups were calculated using one-way ANOVA analysis. * indicates significant differences between normal-weight group and moderately obese group (*p* < 0.05); ^£^ indicates significant differences between normal-weight group and morbidly obese group (*p* < 0.05); ^§^ indicates significant differences between moderately obese group and morbidly obese group (*p* < 0.05); ^#^ indicates significant differences with respect to normal liver (*p* < 0.05); ^‡^ indicates significant differences with respect to moderately obese group with simple steatosis (*p* < 0.05); ^†^ indicates significant differences with respect to moderately obese group with steatohepatitis (*p* < 0.05).

**Table 2:** Accuracy of the miR122 biomarker in the population studied.

Histological Features	AUROC	Cut-Off	Sensitivity (%)	Specificity (%)	PPV (%)	NPV (%)	LR+	LR−
**NAFLD ^a^**	0.82 (0.74–0.90)	0.0045	96.6	54.7	41.6	98.0	2.13	16.14
		**0.0147**	83.1	69.8	47.8	92.5	2.75	4.12
		0.1202	35.6	90.6	55.7	80.8	3.77	1.41
**Hepatocellular Ballooning ^b^**	0.76 (0.66–0.85)	0.0045	97.1	37.2	34.0	97.4	1.55	12.64
		**0.0693**	67.6	74.4	46.8	87.3	2.64	2.30
		0.2738	29.4	93.6	60.5	79.9	4.59	1.33
**Lobular Inflammation ^c^**	0.76 (0.66–0.85)	0.0045	97.1	37.2	34.0	97.4	1.55	12.64
		**0.0693**	67.6	74.4	46.8	87.3	2.64	2.30
		0.2738	29.4	93.6	60.5	79.9	4.59	1.33

^a^ Represent the performance for discriminating NAFLD from control cases; ^b^ Represent the performance for discriminating hepatocellular ballooning from non-hepatocellular ballooning; ^c^ Represent the performance for discriminating lobular inflammation from non-lobular inflammation. The three optimum cutoff values were selected by obtaining a first cutoff of high sensitivity (>90%), a second cutoff which included the best combination of sensitivity and specificity according to the Youden index (in bold), and a third that prioritized specificity (>90%). AUROC, area under the curve of receiver operating characteristics; LR+, positive likelihood ratio; LR−, negative likelihood ratio; NAFLD, non-alcoholic fatty liver disease; NPV, negative predictive value; PPV, positive predictive value.

## Supplementary Materials

Supplementary materials can be found at www.mdpi.com/1422-0067/17/10/1620/s1.
