# Supplementary material for: miR33a/miR33b* and miR122 as Possible Contributors to Hepatic Lipid Metabolism in Obese Women with Nonalcoholic Fatty Liver Disease
Source: Int J Mol Sci. 2016 Sep 24;17(10):1620. doi: 10.3390/ijms17101620 (PMC5085653; doi:10.3390/ijms17101620)
Supplement: Supplementary file 1 [file ijms-17-01620-s001.pdf]

# Supplementary Materials: miR33a/miR33b* and miR122 as Possible Contributors to Hepatic Lipid Metabolism in Obese Women with Nonalcoholic Fatty Liver Disease

**Teresa Auguet, Gemma Aragonès, Alba Berlanga, Esther Guiu-Jurado, Andreu Martí, Salomé Martínez, Fàtima Sabench, Mercé Hernández, Carmen Aguilar, Joan Josep Sirvent, Daniel del Castillo and Cristóbal Richart**

**Table S1.** Correlation between circulating miR122 and mir33b* expression and anthropometrical, biochemical, and liver histological variables in the population studied.

| Variables | miR122 | | miR33b* | |
|---|---|---|---|---|
| | *r* | *p*-Value | *r* | *p*-Value |
| Age (years) | 0.220 | 0.020 | 0.249 | 0.008 |
| BMI (kg/m²) | 0.101 | 0.292 | 0.459 | <0.001 |
| Glucose (mg/dL) | 0.430 | <0.001 | 0.214 | 0.025 |
| Insulin (mUI/L) | 0.150 | 0.138 | 0.111 | 0.274 |
| HDL-C (mg/dL) | −0.305 | 0.001 | −0.276 | 0.004 |
| LDL-C (mg/dL) | −0.129 | 0.195 | 0.031 | 0.752 |
| Triglycerides (mg/dL) | 0.133 | 0.172 | 0.279 | 0.004 |
| AST (U/L) | 0.367 | <0.001 | 0.203 | 0.046 |
| ALT (U/L) | 0.351 | <0.001 | 0.131 | 0.179 |
| Steatosis | 0.017 | 0.880 | −0.143 | 0.187 |
| Lobular inflammation | 0.225 | 0.017 | 0.123 | 0.193 |
| Ballooning | 0.200 | 0.035 | 0.113 | 0.232 |

ALT, alanine aminotransferase; AST, aspartate aminotransferase; BMI, body mass index; HDL-C, high-density lipoprotein cholesterol; LDL-C, low-density lipoprotein cholesterol. The strength of association between variables was calculated using Pearson's *r* correlation test. Bold numbers indicate statistically significant correlations (*p*-value < 0.05).

**Table S2.** Binary logistic regression analysis for hepatocellular ballooning.

| Variables | *B* | *Exp (B)* | *95% CI* | *p*-Value |
|---|---|---|---|---|
| Log miR122 | 0.784 | 2.19 | 1.20–3.99 | 0.010 |
| ALT | 0.032 | 1.03 | 1.01–1.06 | 0.008 |

Backward regression analysis of 112 patients. Nagelkerke $R^2 = 0.373$; $p < 0.001$. Variables entered in the model: Log miR122, age, BMI, HDL-C, triglycerides, AST, and ALT.
